# Current Controversies and Challenges on BRAF V600K-Mutant Cutaneous Melanoma

**DOI:** 10.3390/jcm11030828

**Published:** 2022-02-04

**Authors:** Alessandro Nepote, Gianluca Avallone, Simone Ribero, Francesco Cavallo, Gabriele Roccuzzo, Luca Mastorino, Claudio Conforti, Luca Paruzzo, Stefano Poletto, Fabrizio Carnevale Schianca, Pietro Quaglino, Massimo Aglietta

**Affiliations:** 1 Department of Oncology, University of Turin, 10124 Torino, Italy; alessandro.nepote@ircc.it (A.N.); luca.paruzzo@ircc.it (L.P.); stefano.poletto@ircc.it (S.P.); fabrizio.carnevale@ircc.it (F.C.S.); massimo.aglietta@ircc.it (M.A.); 2 Division of Medical Oncology, Experimental Cell Therapy, Istituto di Candiolo, FPO- IRCCS, str. Prov.le 142, km 3.95, 10060 Candiolo, Italy; 3 Dermatology Clinic, Department of Medical Sciences, University of Turin, 10124 Turin, Italy; gianluca.avallone2@gmail.com (G.A.); fcavallo93@gmail.com (F.C.); roccuzzo.gabriele@yahoo.it (G.R.); lucamastorino02@gmail.com (L.M.); pietro.quaglino@unito.it (P.Q.); 4 Dermatology Clinic, Maggiore Hospital of Trieste, 34125 Trieste, Italy; claudioconforti@yahoo.com

**Keywords:** cutaneous melanoma, BRAF mutation, BRAF V600K, target therapy, immunotherapy

## Abstract

About 50% of melanomas harbour a BRAF mutation. Of these 50%, 10% have a V600K mutation. Although it is the second most common driver mutation after V600E, no specific studies have been conducted to identify a clinical and therapeutic gold standard for this patient subgroup. We analysed articles, including registrative clinical trials, to identify common clinical and biological traits of the V600K melanoma population, including different adopted therapeutic strategies. Melanoma V600K seems to be more frequent in Caucasian, male and elderly populations with a history of chronic sun damage and exposure. Prognosis is poor and no specific prognostic factor has been identified. Recent findings have underlined how melanoma V600K seems to be less dependent on the ERK/MAPK pathway, with a higher expression of PI3KB and a strong inhibition of multiple antiapoptotic pathways. Both target therapy with BRAF inhibitors + MEK inhibitors and immunotherapy with anti-checkpoint blockades are effective in melanoma V600K, although no sufficient evidence can currently support a formal recommendation for first line treatment choice in IIIC unresectable/IV stage patients. Still, melanoma V600K represents an unmet medical need and a marker of poor prognosis for cutaneous melanoma.

## 1. Introduction

Life expectancy in metastatic cutaneous melanoma has changed dramatically in the last decade, after the approval of target therapies and immune checkpoint inhibitors [1]. Target therapies with BRAF/MEK inhibitors have been demonstrated to improve overall survival (OS) and progression free survival (PFS) in metastatic cutaneous melanoma, reaching almost 50% of metastatic patients, due to the high BRAF mutation’s incidence rate, with a very tolerable toxicity profile for patients [2]. Almost 50% of cutaneous melanomas harbour a BRAF mutation [3]. BRAF is a serine/threonine protein kinase that, activating MAPK and ERK signalling, is involved in cell proliferation [4]. The most known mutation (80% of cases) is a substitution of the valine residue at position 600 by a glutamate (V600E) through the mutation of a single nucleotide GTG to GAG. A substitution of the valine residue at position 600 by a lysine through a two nucleotides substitution (GTG to AAG) has been reported as the second most frequent mutation (V600K, 5–30% of cases) [3]. Multiple rare V600 mutations have been described in melanoma (V600D/V600R), although evidence is very limited in terms of clinical characteristics and benefits from target therapy with BRAF/MEK inhibitors [5]. Alternatively, some specific clinical and epidemiological features have been related to the most common V600 genotype, even though most studies do not stratify and elaborate data on OS and PFS according to mutation subtype [5]. The aim of our review is to analyse the open controversies regarding V600K-mutant cutaneous melanoma in different fields: epidemiology, biology, clinical features and response to therapy.

## 2. Epidemiology

Melanoma BRAF V600K at least accounts for 10% of all BRAF mutated melanomas, being the second more common genotype after V600E [3]. However, different percentages have been observed with respect to ethnicity (Table 1). BRAF mutation seems to be more frequent in the Caucasian population, with a higher incidence of V600K mutated melanomas. In Kaori Sakaizawa et al., only 20–30% of melanomas were BRAF mutated and less than 5% were V600K, suggesting that the difference is attributable to the lower incidence of non-chronic sun damage melanoma among Asians [6].

## 3. BRAF Mutation Testing

BRAF mutation testing is routine and it is mandatory for all patients with advanced melanoma, due to its impact on therapeutic decision making [7]. In the last years, several techniques have been developed, including immunohistochemistry (IHC), Sanger sequencing and Next Generation Sequencing (NGS) [7]. IHC has a good profile in terms of sensitivity and specificity, but is not able to detect other V600 mutations, such as V600K [7,8]. Mostly, IHC is indicated as a screening test in specific contexts and needs to be confirmed afterwards by NGS or a PCR- based approach [7]. NGS requires more technology and specific knowledge from healthcare workers, but it allows us to study other gene mutations, such as NRAS and c-KIT [7].

Liquid biopsy is arising as a promising technique for detecting BRAF mutation and for monitoring tumour response to therapy [9]. A meta-analysis by Ye et al., pooling several studies on different tumour types, including melanoma, has reported a sensitivity of 68% and a specificity of 98% for detecting BRAF mutation from a plasma sample [9]. 

## 4. Clinical and Dermatological Features

V600K melanomas have shown to have clinical and dermatological peculiarities from others V600 mutated melanomas. In a multivariate analysis on 308 Australian patients, the melanoma V600K subgroup demonstrated a higher prevalence of patients with older age and objectively appreciable chronic sun-damaged (CSD) skin at dermatological examination, compared to melanoma V600E [10]. The most common primary site for melanoma was the trunk for both genotypes (V600E 38% vs. 41% V600K), although in melanoma V600K the head and neck regions were more frequently affected (33% V600K vs. 11% V600E, respectively) [10]. No differences were found between the proportion of occult melanoma in the two subgroups [10]. In melanoma V600K, the disease-free interval from diagnosis of primary melanoma to the first distant metastasis appeared to be shorter (17.4 months) compared to V600E melanoma (39.2 months), with no significant differences in terms of survival [10]. NGS analysis of 446 melanomas confirmed the higher incidence of V600K mutation in male gender, old age (>60 years) and primary tumour of head, neck, or upper back, with a history of chronic sun exposure and damage. No difference was found between the cutaneous melanomas and metastatic melanomas of unknown origin [11]. In a study on Turkish patients no clinicopathological differences were found between V600E and V600K, although a trend between V600K and male gender/older age and primary tumour of head/neck was found, not reaching statistical significance, probably due to the small sample size [12]. On the other hand, BRAF mutated patients had higher rates of necrosis in primary site and lymphovascular invasion than BRAF wild-type (WT) melanoma patients [12]. Melanoma V600K seems to be associated with high CSD. CSD is linked to chronic ultraviolet (UV) exposure, and solar elastosis has mostly been used as a marker to measure CSD levels, distinguishing between high-CSD and low-CSD melanoma [10,13]. In the World Health Organization (WHO) Classification of Skin Tumours (2018) low-CSD melanomas include superficial spreading melanomas, while high-CSD melanomas incorporate the lentigo maligna subtype and desmoplastic melanomas [13].

Notably, in a prospective study, the proportion of V600K mutations was higher in high-CSD (defined as lesions diagnosed at 55 years of age or older, on the head and neck/shoulder region or dorsal surfaces of hands and feet with high sun exposure levels) than in low-CSD cases [14]. CSD tumours express increased levels of PD-L1 and high-CSD melanoma has been demonstrated to have a higher mutational load than low-CSD: these data could explain why melanoma V600K seems to be more sensitive to immunotherapy than target therapy [14]. BRAF V600 mutated melanomas have shown specific dermoscopic features compared to wild type (WT) melanomas [15]. Notably, blue-grey peppering and white-scar areas were both observed more frequently in MAPK mutant melanomas (either NRAS or BRAF) than in WT [15,16]. These two features are related to histological regression, which has been also associated with MAPK mutant melanomas, supporting the above-mentioned findings. Another dermoscopic trait, the blue-white veil, has been identified as a strong predictor of the presence of BRAF mutation in melanoma [15,17]. Even in dermoscopy, melanoma V600K seems to be characterized by specific features: although studies with big-sized samples have not been performed yet, in Ponti G. et al., three out of four BRAF mutated V600K patients had dermoscopic features such as irregularly distributed globules, blue-grey blotches and blue-white veil, and just in one case peppering was present [18,19]. Although these are preliminary findings, the presence of an alternative pathway of regression different from peppering may be a consequence of the higher aggressiveness and the increased growth rate of V600K melanoma. Alternative dermatoscopic features could be addressed to a different interaction mechanism between melanoma V600K and tumor-infiltrating lymphocytes (TILs), either in terms of quantity or in terms of cells subtype (CD8+/CD4+, Treg FOXP3+), although no formal direct study has been conducted in this regard [20,21,22].

## 5. Biological Identity of Melanoma V600K

As for V600E, BRAF V600K mutation in melanoma promotes a strong activation of the MAPK and ERK pathway, stimulating cell survival and proliferation [23]. Recent preclinical studies have suggested how, biologically, melanoma V600K has its own peculiar features. In 2017 Yuanyuan Li et al. detected how in V600K melanoma the KIT expression gene and c-KIT protein were up-regulated compared to melanoma V600E. Although the significance is unclear, c-Kit is involved in melanogenesis and it may also contribute to melanoma progression and proliferation. Additionally, mir-222 was downregulated and it is implicated in the expression of the KIT gene as an inhibitor, altering metabolic signals [23]. In V600K melanoma, many pro-apoptotic regulators were downregulated as Caspase-7, Bid and Bak, suggesting another mechanism of escape of V600K tumours from apoptosis and promoting cell survival [23]. Melanoma V600K was associated with a lower expression of dual-specificity phosphatase (DUSP6), a transcriptional target of the ERK pathway involved in feedback regulation and reflective of ERK activation, whereas PIK3B expression was higher, as well as tumour mutational load [24]. ERK is a transcriptional factor downline of MAPK signalling that incites a strong stimulus on cell proliferation. PI3KB is part of the PI3K-AKT pathway: when overexpressed, it inhibits apoptosis, promoting cell survival [24]. However, an analysis of molecular expression of high-CSD melanoma, which is known to be correlated with BRAF V600K, has outlined an increment in NF-1 and TP53 mutations, as well as an increase in tumour mutational load compared to melanoma V600E [24]. Research on melanoma has shown how PFS and OS showed either a strong trend or significantly better outcomes as TMB (tumour mutational burden) increased when treated with immunotherapy [25].

## 6. Therapeutic Effects of BRAF/MEK Inhibitors and Immunotherapy on Melanoma V600K

Over the last decades several drugs have been approved for IIIC-IV unresectable/metastatic melanoma, including multiple combinations of BRAF/MEK inhibitors (dabrafenib + trametinib, vemurafenib + cobimetinib and encorafenib + binimetinib) and immunotherapeutic agents, such as nivolumab, pembrolizumab and ipilimumab (Table 2). Although V600K melanoma patients were enrolled in registrative clinical trials, data for this specific subgroup were not or were only partially available, due to V600K rarity and small sample size and to its controversial role as a prognostic and predictive value for response to treatment. This lack of direct data corresponds to an unmet medical need in terms of best and first treatment choice for these patients. In the COMBI-d + COMBI-v trial, 69 patients out of 563 patients enrolled were V600K BRAF mutated, although clinical outcomes for this subgroup were not reported. In a multivariate analysis BRAF V600K was associated with a lower PFS, compared to BRAF V600E. Data on OS did not reach statistical significance [26]. In a coBRIM study, 495 patients (56 BRAF V600K mutated) were analysed in terms of outcomes comparing vemurafenib + cobimetinib vs. single agent vemurafenib; multivariate analysis showed the treatment effect was similar in patients regardless of which BRAF mutation was present [27]. Reported median OS and PFS for V600K patients assigned to combination treatment (24.1 and 12.4 months, respectively) were in line with data for V600E patients (21.9 and 10.6 months), although statistical significance was reached only in the second subgroup, probably due to the greater sample size [27]. In the COLUMBUS study, 577 patients were randomly assigned to either encorafenib + binimetinib arm or single agent encorafenib or single agent vemurafenib. Out of 577 patients, 64 were BRAF V600K mutated and were assigned homogeneously in each treatment arm. The combination encorafenib + binimetinib seemed to be more effective than vemurafenib both in regards to PFS (HR 0.27, 0.11–0.68) and OS (HR 0.31, 0.13–0.74) [28,29]. None of the above-mentioned studies has reported separately OS and PFS for V600K BRAF mutated patients, although both COLUMBUS and COMBI-d + COMBI-v trials confirmed its negative prognostic value. Evidence on which combination is more effective is not provided, even though the most encouraging data supported encorafenib + binimetinib (median OS was 33.6 months: 95% CI 24.4–39.2) [26,27,28,29]. In KEYNOTE-006, 837 patients were randomly assigned to pembrolizumab every two weeks or every three weeks or ipilimumab every 3 weeks for 4 doses. V600K BRAF mutated melanoma patients were not analysed as a single subgroup [30] (Table 2). Similarly, in CheckMate-067 (nivolumab + ipilimumab vs. single agent nivolumab vs. single agent ipilimumab) data on specific OS and PFS for BRAF V600K melanoma are not reported, although results seemed promising for BRAF mutated melanoma with better OS and PFS (5-Y-OS 60% vs. 48% and 5-Y-PFS 38% vs. 35%) compared to WT melanoma, especially in the nivolumab + ipilimumab arm [31]. One of the last frontiers for melanoma therapy is the triple therapy strategy, consisting of BRAF inhibitors + MEK inhibitors + immune checkpoint inhibitors. In KEYNOTE-022, a phase two clinical trial confronting pembrolizumab + dabrafenib + trametinib vs. dabrafenib + tramenitib + placebo, out of 120 enrolled patients, 19 were V600K mutated and homogeneously assigned to both treatment arms. Despite clear evidence of the benefit of the triplet therapy (2-Y-PFS 41% vs. 16.3%, 2-Y-OS 63.0% vs. 51.7%), results were not stratified for BRAF mutations subgroups [32]. Likewise, in IMspire150, a randomised phase 3 comparing atezolizumab, vemurafenib and cobimetinib (atezolizumab group) or placebo, vemurafenib and cobimetinib, results were not stratified according to BRAF mutation subgroups [33]. In IMspire150, 56 patients out of 514 were BRAF V600K mutated (27 in the treatment arm and 29 in the placebo group). Both studies reported positive data for the triplet therapy and even if PFS and OS for the melanoma V600K subgroup have not been studied, the equal distribution of patients to both arms (treatment vs. placebo) both in KEYNOTE-022 and in IMspire150 may suggest the existence of a measurable advantage in terms of survival and progression with triplet therapy for these patients [32,33]. In any case, from these data, it is not feasible to determine if a real benefit exists for the melanoma V600K subgroups or if the reported results are tied to the most numerous subgroup (V600E) or to other factors, such as PDL-1 expression, that may consistently influence all the findings. Inês Pires da Silva et al. tried to compare metastatic melanoma V600K and V600E in terms of OS and PFS after first line treatment with BRAF-inhibitor (BRAFi only agent or BRAFi + MEKi) [24]. The sample comprised 93 patients (78 V600E, 15 V600K) and, despite statistical significance not being reached, there was a worse trend in PFS (5.7 months vs. 7 months) with no V600K patient free from progression after 9 months and a lower complete response rate (0% vs. 10%), yet no differences in OS [24]. Additionally, some limitations of the study were due to the small sample size and to the different distribution of treatment schedules: only 20% of V600E patients were treated with BRAFi + MEKi, while 33% of V600K patients benefited from combination therapy [24] A second cohort (n = 103, 84 V600E vs. 19 V600K) was analysed after receiving a PD-1 agent (nivolumab or pembrolizumab) as first line or as second line after BRAFi ± MEKi; PFS was longer in V600K melanoma patients (median 19 vs. 2.7 months), whereas the prolonged OS for V600K did not reach statistical significance (20.4 vs. 11.7 months) [24]. Recently, the RELATIVITY-047 (relatlimab + nivolumab vs. single agent nivolumab) trial has demonstrated a strong advantage in PFS of the combination of anti-LAG3 + anti PD-1 on single agent anti PD-1, with an excellent toxicity profile, promising to be the future first line of treatment for metastatic melanoma. Although no clinical outcomes for melanoma V600K were reported, no difference in terms of PFS were found between melanoma WT and BRAF mutated melanoma, suggesting that BRAF mutated melanoma patients could also benefit from it [34].

## 7. Discussion

V600K Melanoma patients account for 10% of all BRAF mutated melanoma [3]. The literature has shown how V600K melanomas represent a specific population with its own peculiarities, but still the approach and the therapeutic path are mostly comparable to the ones for other genetic subtypes of cutaneous melanomas [10]. Even though data on V600K melanomas are partial and incomplete, there are some points of agreement among the different studies mentioned above. Epidemiologically, BRAF mutated V600K melanomas seem to be more common in older and male patients, often with a history of CSD [10,14]. In addition, a history of CSD and sun exposure may explain the higher incidence of primary tumour in the head, neck and upper dorsal regions, compared to melanoma V600E [10,14]. BRAF mutated melanoma arose more frequently in the Caucasian population compared to other ethnicities and it may be also linked to the effect of CSD on the fair skin phototype [10,35]. In the Asian population, BRAF melanoma and, proportionally, V600K melanoma seem to be a rarer entity [36,37]. The effect of UV could partially explain the different biological background between melanoma V600E and V600K: furthermore, it seems to justify the major rapidity and aggressiveness of the latter [14]. Preclinical data have highlighted a completely different mutational background in melanoma V600K with the overexpression of c-KIT, the inhibition of multiple proapoptotic pathways and a reduced dependence on the ERK/MAPK pathway [23,24,25]. The overexpression of c-KIT could justify the clinical aggressiveness of melanoma V600K, while the lesser dependence on the ERK/MAPK pathway and the expression of alternative signalling (including PI3K-AKT) may explain why melanoma V600K seems to have a different response to BRAF inhibitor and immunotherapy compared to melanoma V600E [23,24,25]. Melanoma V600K carries a higher mutational load compared to V600E melanoma: in Pires Da Silva et al., mutational load was determined in two cohorts by NGS and The Cancer Genome Atlas (TCGA) whole exome sequencing (WES), respectively, and mutational load was calculated as number of mutations per sample [24]. The higher mutational load may be the key to clarifying why immune checkpoint inhibitors such as nivolumab or pembrolizumab seem to be more effective in V600K melanoma, although further studies are needed to shed light in this regard [24]. No specific prognostic or predictive factor to response to treatment is known for melanoma V600K, although there is a large consensus that for these patients prognosis is worse than for melanoma V600E [10,24]. The combination of BRAF and MEK inhibitor has been demonstrated to be more effective than a single agent BRAF inhibitor even though no direct studies have been conducted to identify which combination would be more adequate for V600K melanoma patients [27,28,29]. Updated results presented at the 2021 ESMO Congress about the COLUMBUS clinical trial have confirmed a continued long-term benefit for melanoma V600K patients for the encorafenib + binimetinib combination compared to single agent vemurafenib at five years [38]. The higher expression of the PI3K pathway in melanoma V600K may be taken into consideration as a future therapeutic target. Alpelisib, an inhibitor of PI3KCA recently approved for metastatic breast cancer by the FDA [39], may also be considered in the treatment of melanoma: however, further knowledge should be acquired on the underlying working mechanisms with respect to this tumour. Current evidence is insufficient to support a formal recommendation using single agent anti PD-1 or anti PD-1 and anti CTLA-4. Melanoma V600K patients clearly benefit from immunotherapy both in terms of PFS and OS; however, data elaborated from registration studies do not permit the quantification of the real impact of immunotherapy, specifically for V600K [30,31]. Results from Pires de Silva et al. are encouraging, even though the small sample size and the short follow up time do not permit us to draw any definitive conclusions [24]. The combination with anti PD-1 + anti-BRAFi/MEKi as a first line therapy might be another promising strategy, considering the risk of not having a real second line therapy in case of progressive disease [32,33]. Recent findings published on anti LAG-3 agent relatlimab have opened a completely new perspective on metastatic melanoma treatment with really encouraging data in terms of PFS that, if confirmed in OS, may lead to a new standard of treatment both for WT and BRAF mutated melanoma [34]. In any case, the need for having specific data on the V600K melanoma population is urgent and may have a strong impact on these patients’ prognoses. Future studies should be conducted to clarify the different biology of melanoma V600K, in order to show the most appropriate clinical and therapeutic pathways for these patients.

## 8. Conclusions

To date, the literature has shown that melanoma V600K has its own biological features, which should not be overlooked. However, further investigation on V600K melanoma is needed. Melanoma V600K represents a specific subgroup of cutaneous melanoma, with a high prevalence among Caucasian, male and elderly patients, often with a history of CSD. Melanoma V600K has been demonstrated to be significantly more aggressive and rapid in progression than melanoma V600E with inclusive or partial data in terms of OS and PFS after receiving either one of the principal therapies approved for unresectable/metastatic melanoma (immunotherapy (PD/PDL-1 agent, CTLA4 agent) or BRAFi ± MEKi). Both target and immunotherapy seem to be effective for V600K melanoma, although no gold standard has been identified yet. Future studies should clarify which therapeutic strategy may be more effective for this specific patient subgroup. In addition, prognostic factors on treatment response could be usefully investigated.

**Table 1:** Patient characteristics among different studies.

Author/Year	Country	Ethnicity	n. Patients	n. BRAF Mutated	n. V600K (%)	Sex (M/F) and Age ^a^ (Years)	Site and Number of Primary Lesion ^a^	High CSD Number of Patients (%) ^a^
Jin SA et al., 2013 [36]	Korea	Asian	202	24 (12%)	2 (8%)	M, 72 M, 76	Scalp, 2 (100%)	1 (50%)
Lyle et al., 2016 [35]	Australia	Caucasian	713	269 (38%)	35 (22%)	-M/F ratio from 1.6 to 2.7 ^c^ -median age from 60 to 69 ^c^	- ^b^	- ^b^
Menzies et al., 2012 [10]	Australia	Caucasian	308	143 (46%)	27 (19%)	-Sex ^e^ -median age 61	-Extremity, 3 (11%) -Trunk, 11 (41%) -Head and neck, 9 (33%)	12 (75%)
Si L. et al., 2012 [37]	China	Asian	438	110 (25.5%)	3 (2.7%)	- ^b^	-^b^	0
Can et al., 2018 [12]	Turkey	Turkish	61	34 (55.7%)	11 (32.4%) (M 6, F 5)	-median age 74	-Extremity, 1 (20.0%) -Head and neck, 6 (54.4%)	- ^b^
Sakaizawa et al., 2015 [6]	Japan	Asian	171	52 (30.4%)	3 (5.8%)	- ^d^	- ^d^	1 (33%)
Sanna et al., 2020 [14]	Sweden	- ^b^	72	35 (49%)	7 (20%)	- ^d^	-Head and neck/shoulder region or hands and feet, 4	4 (57)

CSD: chronic sun-damaged; n.a.: not applicable; n.e.: not evaluated; ^a^: reported data just for Melanoma V600K; ^b^: not reported or evaluated in the study; ^c^: different cohort; n.: number enrolled between 2009–2013; ^d^: data are not stratified for V600K, but just reported for BRAF mutated or following other criteria; ^e^: non statistically significant.

**Table 2:** Overall survival and progression free survival reported among the studies for melanoma patients and for melanoma V600K patients treated with BRAFi ± MEKi and/or immunotherapy (anti-PD/L-1 and/or anti-CTLA-4).

Study	Treatment	Stage AJCC 8th Ed.	n. BRAF Mutated (%)	n. V600K (%)	OS for All Patients (%/Months) †	PFS for All Patients (%, Months) †	OS for Melanoma V600K Subgroup §	PFS for Melanoma V600K Subgroup §
COMBI − d + COMBI v, 2019 [26]	dabrafenib + trametinib	IIIC-IV	563 (100)	69 (12)	-5Y-OS 34%, 25.9 months	-dabrafenib + trametinib 5Y-PFS: 19%, 11.1 months	V600E vs. V600K HR 0.77 (0.55–1.06) ^a^	V600E vs. V600K HR 0.65 (0.49–0.87) ^a^
COLUMBUS, 2018 [28,29]	- encorafenib + binimetinib - encorafenib only agent - vemurafenib only agent	IIIB-C IV	577 (100)	-encorafenib + binimetinib: 22 (11) -encorafenib only agent: 19 (10) -vemurafenib only agent: 23 (12)	-Encorafenib + Binimetinib: OS 33.6 months (2y-OS 57.6%) -Encorafenib only agent: OS 23.5 months (2y-OS 49.1%) -Vemurafenib only agent: OS 16.9 months (2y -OS 43.2%)	-encorafenib + binmetinib PFS: 14.9 months -encorafenib only agent PFS: 9.6 months -vemurafenib only agent PFS: 7.3 months	Encorafenib + binimetinib vs. vemurafenib only agent HR for OS in V600K subgroup 0.31 (0.13–0.74) ^b^	Encorafenib + binimetinib vs. vemurafenib only agent HR for PFS in V600K subgroup 0.27 (0.11–0.68) ^b^
coBRIM, 2016 [27]	-cobimetinib + vemurafenib (Arm 1) -vemurafenib + placebo (Arm 2)	IIIC-IV	495 (100)	Arm 1. 32 (12) Arm 2. 24 (10)	-cobimetinib + verurafenib 22.3 months (2y-OS 48.3%) -vemurafenib + placebo 17.4 months (2y-OS 38.0%)	-cobimetinib + vemurafenib PFS: 12.3 months -vemurafenib + placebo PFS: 7.2 months	median OS cobimetinib + vemurafenib in V600K: 24.1. months median OS placebo + vemurafenib in V600K: 16.7 months HR V600K median OS cobimetinib + vemurafenib vs. placebo + vemurafenib: 0.79 (0.37–1.69) ^c^	median PFS cobimetinib + vemurafenib in V600K: 12.4 months median PFS placebo + vemurafenib in V600K: 6 months HR V600K median PFS cobimetinib + vemurafenib vs. placebo + vemurafenib: 0,52 (0.27–1.02) ^c^
KEYNOTE 006, 2017 [30]	-pembrolizumab every 3 weeks -pembrolizumab every 2 weeks -ipilimumab	III-IV	307 (37)	- ^d^	pembrolizumab every 3 weeks 2Y-OS: 55% -pembrolizumab every 2 weeks 2Y-PFS: 55% -ipilimumab 2Y-PFS: 43%	-pembrolizumab every 3 weeks 2Y- PFS: 28% -pembrolizumab every 2 weeks 2Y-PFS: 31% -ipilimumab 2Y-PFS: 14%	- ^d^	- ^d^
CHECKMATE 067, 2019 [31]	-nivolumab plus ipilimumab -nivolumab alone (N alone) -ipilimumab alone (I alone)	IIIC-IV	298 (31)	- ^d^	-nivolumab plus ipilimumab 5Y-OS: 52% -nivolumab 5Y-OS: 44% -ipilimumab 5Y-OS: 26%	-nivolumab + ipilimumab 5Y-PFS: 36% -nivolumab 5Y-PFS: 29% -ipilimumab 5Y-PFS: 8%	BRAF mutated vs. WT in nivolumab+ ipilimumab 5y-OS 60% vs. 48% ^e^	BRAF mutated vs. WT in nivolumab+ ipilimumab 5y-PFS 38% vs. 35% ^e^
KEYNOTE 022, 2020 [32]	-pembrolizumab with dabrafenib and trametinib -placebo with dabrafenib and trametinib	IIIC-IV	120 (100)	19 (16)	-pembrolizumab with dabrafenib and trametinib 2Y-OS: 63% -placebo with dabrafenib and trametinib 2Y-OS: 51.7%	-pembrolizumab with dabrafenib and trametinib 2Y-PFS: 41% -placebo with dabrafenib and trametinib 2Y-PFS: 16.3%	- ^d^	- ^d^
IMspire150, 2020 [33]	-atezolizumab, vemurafenib, and cobimetinib -placebo, vemurafenib, and cobimetinib	IIIC-IV	514 (100)	56 (9)	atezolizumab, vemurafenib, and cobimetinib: 2Y-OS 60.4% (median 28.8 months) ^f^ -placebo, vemurafenib, and cobimetinib: 2Y-OS 53.1% (median 25.1 months) ^f^	PFS atezolizumab, vemurafenib, and cobimetinib:15.1 months ^g^ PFS placebo, vemurafenib, and cobimetinib: 10.6 months ^g^	- ^d^	- ^d^
Inês Pires da Silva et al., 2013 [24]	-V600K vs. V600E treated with BRAFi ± MEKi ^h^ (cohort 1) -V600K vs. V600E treated with Nivolumab or Pembrolizumab (cohort 2)	IIIC-IV	BRAFi +MEKi: 93 (100) Nivolumab/Pembrolizumab:103 (100)	BRAFi ± MEKi: 15 (16) Nivolumab/Pembrolizumab: 19 (18)	- ^d^	- ^d^	BRAFi ± MEKi: V600E 20 months vs. V600K 18 months p 0.87 Nivolumab/pembrolizumab: V600K 20.4 months vs. V600E 11.7 months	BRAFi ± MEKi: V600K 5.7 months vs. V600E 7.1 months p 0.15 Nivolumab/pembrolizumab: V600K 19 months vs. V600E 2.7 months
RELATIVITY-047 [34]	-Relatlimab + Nivolumab -Nivolumab single agent	III unresectable- IV	275 (38.5)	- ^d^	- ^d^	Relatlimab + Nivolumab 1y-PFS 47.7%, 10.1 months ^h^ Nivolumab single agent 1-PFS 36,0%, 4.6 months ^h^	- ^d^	BRAF mutated vs. WT: no differences for both arms.

OS: overall survival; PFS: progression free survival; HR: hazard ratio; WT: wild type; NE: non evaluable; Y = years; n.: number. §: if data on OS and PFS for melanoma V600K were not reported/evaluated in the study, we reported any difference analysed in the V600K subgroup. †data on OS and PFS are reported either in percentage (number of patients still alive at 2, 4 or 5 years from start of treatment) or in months (median survival reached in months, according to follow up), when available. ^a^: multivariate analysis of baseline factors (V600K vs. V600E) associated with PFS and OS (HR). Specific data on OS and PFS were not reported. ^b:^ OS and PFS by prespecified subgroups (V600K) according to baseline characteristics for the encorafenib plus binimetinib group versus the vemurafenib group (HR). Data on OS/PFS according to baseline characteristics for the encofenib only agent were not analysed. ^c^: OS and PFS for prespecified group analysis (V600K), reported also in terms of HR. ^d^: not evaluated or reported in the study. ^e^: data reported refer to BRAF mutated (V600E + V600K) and not just for V600K. ^f^: data refer to estimated 2y-OS with Kaplan-Meyer. ^g^: data refer to a median follow up of 18.9 months reached. ^h:^ BRAFi ± MEKi combinations with which patients have been treated are not specified in the study. ^h^: median follow up was 13.2 months.

## Data Availability

The authors confirm that the data supporting the findings of this study are available within the article.
